# The United Nations Convention on the Rights of Persons with Disabilities and Social Work: Evidence for Impact?

**DOI:** 10.3390/ijerph20206927

**Published:** 2023-10-16

**Authors:** Pearse McCusker, Lauren Gillespie, Gavin Davidson, Sarah Vicary, Kevin Stone

**Affiliations:** 1 School of Social and Political Science, The University of Edinburgh, Edinburgh EH8 9LD, UK; pearse.mccusker@ed.ac.uk (P.M.); lauren.gillespie@ed.ac.uk (L.G.); 2 Advanced Care Research Centre, The University of Edinburgh, Edinburgh EH16 4UX, UK; 3 School of Social Sciences Education and Social Work, Faculty of Arts, Humanities and Social Sciences, Queens University Belfast, Belfast BT7 1NN, UK; 4 School of Health Wellbeing and Social Care, Faculty of Wellbeing Education and Languages, The Open University, Walton Hall, Milton Keynes MK7 6AA, UK; sarah.vicary@open.ac.uk; 5 Centre for Lifelong Learning, The University of Warwick, Coventry CV4 7AL, UK; kevin.stone@warwick.ac.uk

**Keywords:** United Nations Convention on the Rights of Persons with Disabilities (UNCRPD/CRPD), social work practice, mental health social work practice, mental health and capacity law reform, human rights, supported decision-making, social model of disability

## Abstract

The United Nations Convention on the Rights of Persons with Disabilities (hereafter CRPD) has provided a radical imperative for the reform of mental health and capacity legislation around the world. The interpretation of the CRPD has been controversial, ranging from the complete abolition of detention, forcible treatment, and substitute decision-making to accepting that elements of these measures need to be retained based on non-discriminatory criteria, additional safeguards, and a comprehensive shift towards supported decision-making. While the potential effects of the CRPD on mental health social work and social work generally are considerable given their shared commitment towards social justice, to date there has been no review of research evidence exploring their relationship. In addressing this knowledge gap, this study held a preliminary discussion with practitioners and academics at the European Association of Social Work Mental Health Special Interest Group in Amsterdam 2022, followed by a scoping literature review on the question: What impact, if any, has the CRPD had on social work practice? The review produced four main findings: impact on legislation; positive impact on practice; limited impact on practice; and impact on social work education and research. In sum, while there were some positive indications of social work and mental health social work practice being influenced by the CRPD, these were scant. Barriers to change included tendencies among some social workers to practise substitute decision-making, in part related to resourcing and policy contexts, and understandings of disability aligned to individualised/medical rather than social perspectives. The results indicate that legal reform on its own is insufficient to impact social work practice, and that realising the potential of the CRPD will necessitate good quality training, as well as improving social workers’ knowledge of the human rights of people with mental impairment.

## 1. Introduction

The United Nations Convention on the Rights of Persons with Disabilities [1] (hereafter CRPD) was adopted in 2006 and aims to protect the rights of all persons with disabilities, including people who experience mental health difficulties. Since that time, the CRPD has gained 164 signatories and 185 ratifications, representing broad agreement across the vast majority of UN member states. Article 3 of the CRPD outlines eight guiding principles that necessitate signatory States Parties make to a paradigm shift in how persons with disabilities are treated.

The need for legislative and societal reform to address the discrimination and inequalities facing people with disabilities, including mental health problems, is well documented. Writing in the US context, Krahn et al. [2] indicate the historical, social, economic, and environmental disadvantages that have excluded persons with disabilities from adequate health provision. They call for this to be addressed through assigning ‘disparity status’, which would support a more targeted and coordinated approach towards reducing health inequities. Specific to mental health, the evidence base is equally troubling, highlighting the prevalence and deleterious impact of poor mental health on populations globally. Motrico et al. [3] (p. 1) categorise mental disorder as ‘one of the greatest public health concerns of our time’ and highlight social factors, including socioeconomic status, employment, housing, etc., as key causes of mental distress, arguing for these to be assigned as targets for change. In this context, the introduction of the CRPD is generally recognised as a much-needed and progressive step in articulating the human rights of persons with disabilities and mental health difficulties. It has provided a baseline from which to explore and refine the conceptual and practical challenges inherent in this field [4]. For some, including Ramos Pozón, the CRPD goes beyond requiring the application of existing human rights and is tantamount to ‘creating new rights, or at least very new ways of seeing common rights’ and differs from other international human rights treaties in going ‘out of its way to describe what exactly is required of State parties’ [5] (p. 13). The impact of the CRPD on how the human rights of persons with disabilities and mental health problems are understood and supported is arguably significant. For example, it is seen as central to exposing and challenging the discriminatory nature of many existing statutes that mandate the erosion of autonomy based on mental health diagnosis [6]. It has also supported more practically orientated initiatives. For example, in a recent study, Gómez et al. [7] consider how quality-of-life indicators might be used to assess the degree to which CRPD Articles may be operationalised. Additionally, the CRPD has been found to influence research activity regarding disability and mental health, albeit this has highlighted significant gaps, particularly around implementation strategies [8]. 

Set against this background, the opportunities the CRPD provides for mental health social work, and social work practice as a whole, are potentially transformative. In terms of values and aims, the alignment between the CRPD and social work is manifest in a shared commitment to upholding human rights and autonomy, promoting equality, social inclusion and social justice, and addressing discrimination related to disability and mental health [9,10]. Both the CRPD and social work practice also share the social model of disability as an organising theory, which requires a move away from locating mental health and disability at the level of the individual to understanding and addressing the systemic barriers (as indicated above) that restrict equal access to human rights [10,11]. This theory is evident in the CRPD, which states ‘that persons with disabilities have the right to recognition everywhere as persons before the law’ (Article 12(1)) and requires countries to ‘recognize that persons with disabilities enjoy legal capacity on an equal basis with others in all aspects of life’ (Article 12(2)). In turn, this means taking ‘appropriate measures to provide access by persons with disabilities to the support they may require in exercising their legal capacity’ (Article 12(3)). It is important to note that Article 12 also specifies that it is necessary to ensure that there are ‘appropriate and effective safeguards to prevent abuse’ and that they ‘respect the rights, will and preferences of the person’ (Article 12(4)). 

The Committee on the Rights of Persons with Disabilities General Comment 1 (hereafter the Committee) is the body that monitors the implementation of the CRPD by the States Parties, and it also publishes interpretations of human rights provisions [12]. The Committee has interpreted Article 12 as necessitating the removal of existing substitute decision-making regimes and replacing them with supported decision-making approaches [13]. Furthermore, Article 14 of the CRPD asserts that all adults have a right to liberty and security, which the Committee has interpreted as meaning detention and forcible treatment based on the diagnosis of a ‘mental disorder’ are discriminatory and should be abolished entirely [14]. 

Taken as read, even without the Committee’s more radical interpretation, these changes require radical legal reform. A debate has centred on the compatibility of retaining some forms of substitute decision-making, detention, and forcible treatment with the CRPD if non-discriminatory criteria are introduced and safeguards increased. Advocates of this position argue that substitute decision-making is needed to protect a person’s right to dignity, safety, and well-being where they cannot, even with support, make a meaningful decision [11,15]. While arguments about interpretation will continue, the CRPD has evidently started a process of a far-reaching reform of mental health and capacity legislation. For example, this is reflected in the emergence of fusion legislation, in which a mental health diagnosis is replaced with capacity as a legal criterion for intervention on the basis that capacity applies to everyone. The argument is that this removes the discriminatory link between mental health diagnosis and intervention, thereby complying with Article 14. The Mental Capacity (Northern Ireland) Act 2016 is the only example of enacted fused legislation, but other jurisdictions may follow. There appears to be more consensus on the role of supported decision-making as a key approach through which to achieve equality of legal capacity, as sought by Article 12. This is evident in the emphasis placed on supported decision-making in proposals for legal reforms [11,16]. In practical terms, legislating for supported decision-making should speed up the development of approaches that enable people with disabilities and their support networks to express and give effect to their will and preferences. Examples include independent advocacy services, decision-making supporters, and powers of attorney, among others [16]. The CRPD’s focus on human rights also extends to addressing the structural factors that impact mental health and disability, and this is also beginning to shape legal change. For example, the recent Scottish Mental Health Law Review’s [16] recommendations are predicated on putting economic, social, and cultural rights at the heart of any new statute. This is an acknowledgement of the social determinants of mental impairment, the range of intersectional barriers affecting people with mental health difficulties and disability, and the need for more systemic responses that address issues such as poverty and racial discrimination. 

While the authors of this paper strongly support the direction of this reform agenda, any impact the CRPD has had beyond the legal domain remains unclear. For social work, and mental health social work especially, there are many unanswered questions about the degree to which the principles and provisions of the CRPD have filtered down to the level of day-to-day practice. In seeking further discussion of these important issues, this paper explores research evidence with a view to increase the understanding of the CRPD’s impact on social work. The objectives include an examination of how the CRPD has influenced the knowledge, skills, and values that inform social workers’ practice with service users. What impact it has had on the design and delivery of social services? What effect has this had on service users’ lives and what does it signify in terms of upholding their human rights? 

## 2. Research Design, Materials, and Methods

In adopting an exploration and analysis of existing research evidence on the impact of the CRPD in social work, this study fits predominantly within a theoretical research design typology [17]. It comprised two stages: first, a preliminary discussion among attendees at the European Association for Social Work Research’s (EASWR) Mental Health Special Interest Group (MHSIG) meeting in Amsterdam, April 2022; and following this, a scoping literature review. While the research team was particularly interested in mental health social work, we recognised there was likely to be insufficient evidence to support an exclusive focus on this and consequently, the scope was widened to social work as a whole, resulting in the research question: What impact, if any, has the CRPD had on social work?

### 2.1. Discussion Group

Discussion at the MHSIG was foregrounded by a short presentation given by the lead author, addressing the key provisions of the CRPD and its influence on mental health law. Participants, 21 in total, from 7 countries—Czech Republic, England, Finland, Northern Ireland, Norway, Scotland, Slovenia—were then invited to discuss and reflect on the following questions:

- The extent to which social workers are aware of the CRPD;
- Its impact on social work practice in mental health/other settings;
- The barriers to this and how might they be addressed;
- The potential the CRPD has for social work practice.

The main aim of this preliminary exercise was to stimulate discussion on the subject to explore its relevance and worth as a research study. Participants provided a range of perspectives, reflecting differences in how the CRPD is understood and enacted across the 7 countries. The consensus was, however, that the CRPD represented a valuable lever for change, for example, in supporting the deinstitutionalisation of psychiatric/learning disability hospitals and in campaigning for service user rights. At the practice level, however, there was felt to be limited awareness of the CRPD among social workers and a dearth of evidence for wider impact. As such, participants felt there was a long way to go before the CRPD’s change potential filtered down to the level of social work practice. Despite this, attendees considered the CRPD as potentially transformative for social work and supported the study’s aims to explore the impact further. 

### 2.2. Scoping Literature Review

The research team agreed on a literature review as the best means of undertaking the inquiry, which was subsequently carried out by a doctoral student, with the rest of the research team acting as a supervisory panel. Two additional doctoral students assisted with initial screening, and one member of the supervisory panel acted as a decision maker where there was conflict. The original intent behind the study was to complete a rapid review of existing literature in this area; however, this was changed to a scoping literature review in alignment with the review’s purpose of determining ‘… the scope or coverage of a body of literature on a given topic’ [18] (p. 21) because the evidence base was anticipated to be limited. A scoping review retained the systematic approach the research team sought while making it feasible in the context of the study being unfunded and time-limited; for example, by not requiring a critical analysis of the methodologies of studies selected. In line with research ethics practice regarding literature reviews, ethics approval was not required.

#### 2.2.1. Identifying the Sample

To identify relevant literature, searches were conducted in eight databases: ScienceDirect, PubMed, Social Science Research Network (SSRN), EBSCO, CAB Abstracts (CAB Direct), ProQuest, United Nations Database, and Social Care Online. The search terms used were chosen to capture literature that specifically answered the agreed research question: What impact, if any, has the CRPD had on social work? Terms such as ‘social services’ were included to capture wider references to ‘social work’. Search terms were applied to the title, abstract, and keywords only (see Table 1). 

Results were limited to those published in English. No grey literature was included. Results were uploaded to the online systematic review tool Covidence, which was then used for the study selection stage. In addition to the searches completed above, one study was identified and suggested by a member of the supervisory panel due to its relevance to the research question. Although the database search did not capture this study, it was cited by another source that was included. This indicates that hand searching reference lists would have revealed this source. A full hand search of all reference lists was not completed due to time constraints, which is, therefore, a limitation of this study. The supervisory panel agreed that the additional source should be included in the review, nonetheless. 

#### 2.2.2. Study Selection, Charting the Data, and Summarising and Analysing the Results

All search results were double-screened according to their title and abstract. Where there was a conflict about inclusion at the initial screening stage, one member of the supervisory group resolved these. There was actually a relatively high level of conflict at the initial screening stage that led to a narrowing of focus. Though many sources could be perceived as inherently linking the CRPD with social work due to the broad nature of the social work discipline, papers were excluded unless they explicitly stated that their aims were to explore such a link between the two. For example, some studies that explored the impact of the CRPD within a healthcare setting were excluded, given that the presence of social work was not clear. Full-text screening was then completed and reviewed by the supervisory group. Following the final paper selection, a literature summary table was devised, as recommended by Younas and Ali [19], which captured information on the author(s); year of publication; country of origin; aims/purpose; study population and sample size; method and intervention type/comparator/outcome measures; and key findings related to the research question and summary/notes and emerging themes/ideas (see Supplementary Table S1). The main themes were identified for each source using inductive thematic analysis [20], and sources were then ‘grouped’ by similarity. Four clear overarching themes emerged, as summarised in Section 3, and a scoping review was prepared utilising these themes for the structure [21,22].

#### 2.2.3. Results of the Search and Screening

**Figure 1:**
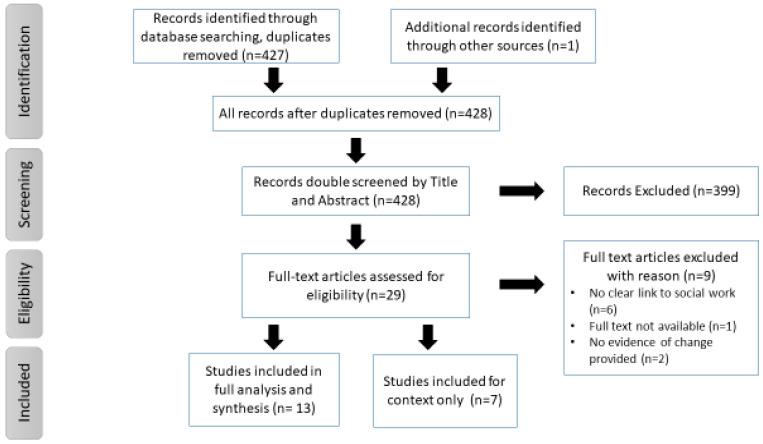
Prisma flow chart of the search and screening results.

The initial database search identified 427 sources after duplicates were removed, including 1 additional source that was identified by the supervisory panel; a total of 428 sources were screened. Following the title and abstract screening, 29 sources were screened by their full text. This screening excluded a further 9 sources, providing a total of 20 for inclusion and data extraction; see the Prisma flow chart in Figure 1. As anticipated, the included publications represent an array of contexts and findings beyond mental health social work.

## 3. Results

Analysis of the studies led to the identification of four main themes: impact on legislation; positive impact on practice; limited impact on practice; and impact on social work education and research. Each of these are now presented.

### 3.1. Impact on Legislation

As indicated, the CRPD directs signatories to alter their policy and practice for persons with disabilities. Because of this, a large portion of analysis since its introduction has been on how countries have approached the crafting of new legislation to align with its tenets. Given the breadth of the term ‘disability’, legislation formulated in this way has implications for those with mental impairment, intellectual disabilities, and/or conditions associated with older age, including dementia. Of the 20 sources included in this review, however, only two discussed how legislative changes in signatory countries have specifically influenced social work practice. These two sources will be summarised more fully, although several of the other sources are utilised to understand the wider changes in this area. 

Campbell et al. [23] examined the role of mental health social workers in four jurisdictions—the UK, Ireland, Victoria (Australia), and Ontario (Canada). The authors investigated how the policies adopted in these locations attempt to deal with the complex issues arising for people with a mental impairment, and in turn, how this translates into social work practice. Campbell et al. [23] point out that, in general, there is now a trend towards supported decision-making and a presumption of legal capacity in many legal jurisdictions. They explain that this change has a direct impact on the role of mental health social workers on the frontline of enacting these policy changes. They acknowledged, however, that despite the overall trend in legislation that endorses supported decision-making, the practicalities of applying it and influencing professional judgement within this process vary widely. 

Campbell et al. [23] summarised the legislation in these four jurisdictions and compared their unique legislative approaches to assessing capacity for decision-making, providing compulsory treatment, and utilising safeguards. They explored the role of social workers within each jurisdiction by comparing the same case study vignette in each setting. This approach sought to illustrate how the legislation was enacted, what emphasis was given to supported decision-making, and what protection was afforded to the individual’s rights. It was clear that social workers had a role in the enactment of the legislation in each location; however, the authors demonstrated that there was a more distinct link to the CRPD in the places where the legislation is recent (such as Northern Ireland’s legislation in 2016 and Victoria, Australia’s legislation in 2014), rather than where it predates the CRPD (such as in England and Wales). The authors provide a link between the CRPD, legal changes within signatory countries, and the provision of mental health social work.

While Campbell et al.’s [23] study indicates some progress, another study based in Lithuania provided a contrasting view. Levickaitė and Mataitytė-Diržienė [24] argue that while the CRPD has influenced some legislative reform in Lithuania, it has not altered social service policy at a local level. The authors specifically looked at the Vilnius municipality in Lithuania in terms of its ability to comply with the obligations under Article 19 of the CRPD, which emphasises deinstitutionalisation and the provision of community-based services. Despite being signed in 2007 and ratified into Lithuanian law in 2010, the authors note that local Lithuanian policy has been slow to progress in line with the CRPD. While national legislation was amended in 2016 to provide some alternatives to total guardianship, the option to implement guardianship still remains. The authors argue that despite this progress within national legislation, there remains an inadequate provision of supported decision-making schemes, community-based services, and suitable housing for those with disabilities. Specific criticism is directed by the authors at the Vilnius Municipality for being ‘unprepared’ to implement changes that align with Article 19 of the CRPD. The authors conclude with recommendations for how to adjust local policy, including a critical observation that one key barrier to tangible change is the lack of adequate funding for new and improved services. 

Without explicitly discussing the provision of social work, several other sources describe law and policy changes that would clearly impact the social work role, given they are discussing the delivery of social services generally. Büschi et al. [25] reviewed legislation and policy in Switzerland regarding people with intellectual disabilities since the introduction of the CRPD. The authors found evidence of positive progress, including more autonomy allotted to spending of benefits. They note that care in the community is increasingly normalised but that the State is ‘bound’ to fund residential care services, making it difficult to fund more progressive alternatives. Davidson et al. [26] compared the legal frameworks for supported and substitute decision-making in Australia, Canada, England and Wales, and Northern Ireland, illustrating how the CRPD had been a powerful facilitator of change. Doyle and Flynn [27] and Flynn [28] reviewed CRPD-related law and policy changes that have occurred in Ireland. This provides context for how CRPD has shaped policy development around capacity and, more recently, how it has supported a policy shift away from the medical model of disability and towards the social model. Flynn [28] highlights this as an important indicator of reform where, because key stakeholders and policymakers have changed their view, the general public’s understanding of disability has begun to shift as well. 

Others have cited case law examples rather than new legislation to evidence how policy has been influenced by the CRPD. Smith [29] explains the landmark case in England, P v Cheshire West and Cheshire Council, where the judge made specific reference to the CRPD in the ruling, which in turn helped to strengthen Deprivation of Liberty safeguards, a set of measures introduced to the Mental Capacity (2005) Act, including a right to legal representation. Sugiura et al. [30] provide a brief but broad overview of changing legislation regarding mental health treatment. They focus on how signatory countries have altered their mental health laws, particularly in relation to involuntary treatment, due to the CRPD. Several countries that have designed legislation to align with the CRPD were noted, including India, Costa Rica, and Peru, amongst others. Examples were also provided of legislation that relies on non-coercive strategies rather than involuntary treatment models, further evidencing a general shift in the direction endorsed by the CRPD. 

Finally, one source examined how the CRPD may hold less predictable consequences for the law, such as for parents with disabilities where there is an assessed risk to the child. Ooi and Loh [31] conducted a case law analysis to explore how parents with disabilities may have additional rights to receive support according to the CRPD. Although the authors’ argument is theoretical only, they demonstrate how the CRPD could have implications for social work with children and families, an area with which it is not usually associated. 

### 3.2. Positive Impact on Practice

Of the twenty sources included in the review, nine directly investigated if and how the CRPD has or could change social work practice. Of these studies, four found evidence of change or potential change, while five concluded there has been very little impact, even in some cases where legislation has been reformed. 

Quejido-Molinero and Miranda-Ruche [32] analysed two case studies from Spain to demonstrate how ethical dilemmas arising since the ‘paradigm shift’ of the CRPD may be addressed. Their examples included one man with an intellectual disability and one couple with severe mental impairment. Both case studies took place within residential care settings, where staff struggled with the tension between allowing the self-determination of the individuals while also looking after their best interests and managing risk. In both examples, staff teams employed a strategy to help them determine a rights-based course of action: one team relied on group supervision to discuss the dilemma and possible solutions, while the other utilised Ethical Reflection Spaces in Social Intervention Services (ERESS). These strategies allowed the staff to vent their concerns and reach an agreement on a course of action, which helped the residents make their own decisions. The outcomes were empowering in each case and avoided the common strategy of compelling (i.e., forcing) a certain decision, which reassured the staff. 

The authors acknowledge that the professionals held anxieties about allowing these decisions, but these fears were ultimately overridden by their determination to protect the rights of the individuals. The professionals’ ability to access group supervision or reflection spaces allowed them to make a coherent plan as a team, thereby alleviating the feeling of personal responsibility for any individual worker. The authors provide a clear example of how the ratification of the CRPD has begun to change the way practitioners consider a person’s legal capacity and, therefore, change the way decision-making is approached for those with disabilities. 

A similar but older example of how the CRPD has impacted practice is provided by Wilkins [33]. Wilkins analysed a case in England where a social worker was involved with a 21-year-old person with autism and was required to balance the desires of the caregivers (parents), the will of the individual, and their professional duty to protect the individual from abuse. Similar to Quejido-Molinero and Miranda-Ruche [32], Wilkins illustrates how social workers often face the difficult task of protecting a person’s well-being while also supporting them to uphold their rights. Wilkins [33] emphasises how the social worker must navigate the competing rights granted by the CRPD, such as the freedom to make autonomous decisions (Article 3) and the right to live free of abuse and exploitation (Article 16). Wilkins points out that even where there is a conflict of rights, the CRPD requires that a person must be made aware of *all* their rights and options (Article 21), a duty that was upheld by the social worker in the case study. According to Wilkins, this approach fostered a trusting relationship between the social worker and the individual, which in turn produced a positive outcome despite the complexity of the situation. While Wilkins only offers one example and makes no direct comparator to practice *before* the CRPD was in place, this piece does provide evidence that it is having some effect on how practitioners approach decision-making in England.

Whereas these first two sources discuss how practice has changed within existing services, Curto and Marchisio [34] provide an account of how the CRPD has changed the structure of one whole service. The authors analysed the 19 Pari Project based in Northwest Italy, which endeavours to support people with disabilities and their families to co-design their own care as they transition into adulthood. Curto and Marchisio describe the 19 Pari Project in-depth and attempt to capture how fundamentally different it is from previous models of intervention. The authors link this project design directly to the ‘paradigm shift’ brought by the CRPD. They suggest that while it is positive, it is merely a first attempt at adopting a drastically different approach in this area. They propose that professionals and researchers alike move away from linear problem solving, which aims to implement interventions based on the individual’s needs, and towards a much wider methodology that changes systems, networks, and communities as a whole. The 19 Pari Project is one example of how service design has changed due to the influence of the CRPD, but the authors note that changes must occur at a much deeper societal level to truly align with the tenets championed by it. 

Lastly, Shik Kim [35] considers the potential impact of the CRPD from a theoretical perspective, articulating how its principles could be harnessed to strengthen rights-based social work practice. He highlights the value of the concept of citizenship in this regard for providing a vehicle to support disability-inclusive practice. 

### 3.3. Limited Impact on Practice

In contrast to the above sources, which evidenced that the CRPD is impacting social work practice, the following five studies found that it has, so far, had relatively limited success in altering practice across several domains, in some instances despite a clear change to legislation. 

One account of this limited success is provided by Harding and Taşcıoğlu [36]. Their ‘Everyday Decisions Project’ investigated how effectively practitioners were providing support for decision-making to people with disabilities in England and Wales. The project consisted of qualitative interviews of 46 people including professionals, supporters, and service users. The study found that practice tended to support an individual’s autonomy to make everyday decisions but generally still reverted to the ‘best interest’ principle for wider, more complex decisions. The authors point out that this tendency is contrary to the principles underlying the Mental Capacity Act (2005) in England and Wales, which endorses supported decision-making and a presumption of capacity, even for complex decisions. 

Harding and Taşcıoğlu’s [36] study was designed to look at supported decision-making in context, not to specifically assess if the CRPD was impacting practice. Despite this, it was included in the review because the authors utilised the standard set by the CRPD as a benchmark and specifically investigated social work practice. The study ultimately concludes that there is evidence of some supported decision-making within daily practice; however, practitioners are slow to adopt this approach more fully, despite the legislation endorsing it. Importantly, the authors note that this occurs where there is a high level of training for staff about the legislation. 

The work by Holler and Werner [37,38] in Israel also looks specifically at social work practice in light of the CRPD. The authors conducted research to assess the attitudes of social work students and current social work practitioners towards supported decision-making. This research was conducted after Israeli legislative reform in 2016, which introduced a limitation on guardianship and endorsed supported decision-making as a priority. This research produced three publications, all with relevance to this scoping review. Two are detailed below, while the final study is included in Section 3.4.

One of the research projects conducted by the pair included semi-structured interviews of 27 Israeli social workers, all currently practising with adults with disabilities. Each participant was asked about their views on guardianship and supported decision-making in practice; their responses were then transcribed and analysed. The aim of this research was to understand practitioner attitudes as well as their decision-making around legal capacity recommendations. 

In one publication, Holler and Werner [37] focused their analysis on the meaning that social workers attach to guardianship and supported decision-making. They found that the interviewees were largely accepting of the use of guardianship, despite their recognition that it includes a high level of restriction. Additionally, the practitioners were sceptical of supported decision-making, and most did not regularly utilise it. The practitioners listed the need to protect people as their primary justification for relying on guardianship. They also noted that despite legislative changes, many service providers still require guardianship to be in place before they will accept a service user. The responses evidence that despite the CRPD’s impact on legislation, individual attitudes, public perception, and service provision are much slower to change. 

In their second piece, Holler and Werner [38] utilised the same body of data collected through interviews to look at how the practitioners make capacity-related decisions. Similar to the above, the authors found that practitioners still link intellectual disability with guardianship by default, despite this being contrary to the CRPD. The research revealed that practitioners made recommendations for guardianship based on the person’s diagnosis, functioning level, and the presence of a supportive family. These factors are incongruent with the presumption of legal capacity, a core tenet of the CRPD. The authors again argue that despite the CRPD influencing legislation in Israel, it has not had a significant impact on the mindset of social workers who are required to uphold such legislation. 

In both pieces, the authors point out that the practitioners demonstrated a lack of awareness of the CRPD and supported decision-making generally and recommend more comprehensive training on it, supported decision-making, and current Israeli law as a means of addressing this lack of awareness. They also argue that clear practice guidelines for assessing capacity could help prevent practitioners from making recommendations based on diagnosis and cognitive functioning alone. 

Another source that reaches similar conclusions is provided by Ruškus et al. [39] regarding Lithuania. The authors surmise that social workers are not yet practising in alignment with the CRPD due to the ‘role conflict’ created when their professional values and local policy clash. To better understand why role conflict occurs in this area and how practitioners react, the researchers conducted semi-structured interviews with six social workers in Lithuania who each had ‘intimate knowledge of the phenomenon of dealing with issues around legal capacity’ (p. 112). 

Their findings confirm that role conflict is a common occurrence for social workers in this area and that this presents a significant barrier to practitioners upholding their professional values. The authors identify three common themes from the workers interviewed: (1) they are susceptible to the ‘pejorative cultural perceptions’ of disability; (2) they lack the ability to advocate effectively for individuals with disabilities; (3) and practitioners feel doubtful of their own mandate, suggesting a lack of competence and awareness (p. 113).

Ruškus et al. [39] go on to explain that practitioners are conflicted by the professional value of human rights and their role requirements under the local legal, policy, and administrative systems in place. This appears to be underpinned by the predominant negative perception of disabilities within the culture, leading to discriminatory legislation and policy. Despite the fact that workers recognised the problematic nature of such cultural views and their own duty to advocate for individuals within such a context, they expressed uncertainty about how to do so effectively and a feeling of powerlessness within the process. Finally, similar to the findings of Holler and Werner [37,38], the social workers demonstrated a general ambivalence about disabilities and a lack of awareness about the rights of such people, including the protection of legal capacity as stipulated by the CRPD. 

The final piece, which suggests the CRPD has *not* greatly impacted social work practice, is provided by Maylea [40]. Writing in an Australian context, Maylea argues that social workers’ approach to involuntary treatment of persons with poor mental health remains largely unchanged, despite the implications of the CRPD for signatories. In a position piece focusing on mental health social work, he argues that despite the professional values that challenge the enforcement of involuntary treatment, there has been a longstanding attitude of amenability to it in practice. He states the two main justifications given for involuntary treatment are that it is in a person’s best interests and that it provides a level of protection to the community. Maylea goes on to dispute these justifications and offers evidence from several disciplines including law, psychiatry, and sociology. He does not provide a substantive alternative to involuntary treatment but does offer examples of rights-based practice techniques, including a mentorship model and the use of advanced directives. Maylea’s paper does not offer a specific comparison or analysis of practice before and after the CRPD was introduced; however, he illustrates how mental health social work has and does endorse involuntary treatment, despite the potential conflict this presents for the CRPD or the social work goal of social justice. 

### 3.4. Social Work Education and Research

Direct practice with individuals does not represent the entirety of the social work profession. Social work is an academic discipline, and, therefore, education and research are important parts of the field. Of the sources identified by the search, three of them address research and education explicitly. 

The first look at education is provided by Werner and Holler, the researchers in Israel who were discussed above. In addition to their semi-structured interviews with current practitioners, Werner and Holler [41] also researched the attitudes of social work students. The authors collected data from 414 undergraduate and graduate social work students in Israel using a questionnaire. The questionnaire measured the students’ attitudes towards guardianship and supported decision-making, the importance they placed on social work goals, and their perception of disability. 

Werner and Holler’s work goes one step further than assessing the current attitude towards guardianship within a sector of the social work profession. They have also gathered data on underlying perceptions of disability (social vs. individual) and professional values (social control vs. social justice) to assess correlation. This helps to unpick why social workers may endorse the new paradigm introduced by the CRPD or reject it. The authors found that social work students *did* support limiting the scope of guardianship but did *not* fully oppose its use. The survey revealed that an individual perception of disability was positively associated with an acceptance of guardianship, whereas a social perception of disability was negatively associated [41] (p. 716). This indicates that where students support the social model of disability and the goal of social justice, their approach to capacity is likely to be more in keeping with the tenets of the CRPD. They conclude that in order to influence social work practice away from guardianship, social work education should focus on changing the orientation of social work students away from the individual model of disability and social control. 

While Werner and Holler [41] included recommendations for educators based on their research with students, others have focused on how the CRPD may already be impacting social work education programmes. Katsui et al. [42] examine an intervention in a social work training programme in Kyrgyzstan. The authors analyse data on the success of an intervention led by the European Union Social Protection Systems Programme. The intervention itself was facilitated by the Finnish Government and provided training to 30 University lecturers between 2017 and 2018. The aim of this training was to change the ‘traditional’ perception of disability within the group of lecturers in order to help the country move towards alignment with the CRPD. 

The authors make plain that disability has historically been viewed negatively in Kyrgyzstan and because of this, persons with disabilities have been segregated from society. Additionally, professionals and individuals in Kyrgyzstan previously lacked awareness of the ‘paradigm shift’ towards the social model of disability and a rights-based approach. To address this, a training programme was implemented to educate social work lecturers on disability rights and the social model of disability. The results of the research by Katsui et al. [42] indicate that perceptions of disability were changed dramatically by the training, leading to more inclusive attitudes and a desire to reform the social service provision within the country. 

This intervention for social work educators is a prime example of the CRPD making a tangible impact in the field. The authors of this study and many of the participants themselves are quick to note that teaching a relatively small group of lecturers is far from sufficient to change an entire nation’s approach; however, it can influence the curriculum, attitudes, and perhaps eventually the practice of social workers in the future. A key factor is that this intervention and the resulting shift may never have occurred without the existence of the CRPD.

Beyond these examples of how students and educators have been impacted by the CRPD, one study discusses the way that it has impacted research within the field. Stevenson [43] argues that the CRPD directs researchers to partner more fully with persons with disabilities. Stevenson suggests that while disability research has historically encouraged the inclusion of people with disabilities, the CRPD established their direct involvement as one of their explicit rights, and, therefore, nullified many of the common reasons for excluding this population.

Stevenson, a social work researcher and educator, describes a project involving young people with Down syndrome as one example of how barriers to inclusive research may be overcome. She asserts that the CRPD requires researchers to double their efforts to include those with disabilities, and she suggests that this may be achieved by adopting a role as an ‘activist researcher’ [43] (p. 38). Here, Stevenson offers an insight into how the CRPD is shaping academic research within the social work field. 

## 4. Discussion

It is evident from this scoping review that the body of research exploring the impact of the CRPD on social work is very limited, and even more so for mental health social work specifically. This aside, this review has provided the first synopsis of this subject. In doing so, it has delineated some of the main challenges facing social work in capitalising on the CRPD’s potential as a lever for change, some of which make for uncomfortable reading for social work practitioners and academics; for example, reflecting a disjuncture between professed values around the social model of disability and practice on the ground. However, it has also highlighted approaches that would appear to represent the kinds of transformative change the CRPD was designed to achieve, which act as helpful signposts to how this might be consolidated and further developed. The principal messages from the review are now discussed.

### 4.1. Legislative Change Alone Does Not Necessarily Translate into Practice

One of the key findings of this review is that while legislation concerning mental health and capacity is changing around the world due to the CRPD, it is not necessarily having a direct impact on social work practice. There is a good deal of research into how the CRPD is influencing legislative reform within the signatory countries. While legislation was not the focus of this review, due to the links between social work and law, and mental health and capacity law in particular, there was an inevitable focus on this area. And, while a full investigation of changing legislation is outside the scope of this project, some clear evidence has been provided by the literature, which has implications for social work. 

Legislation influenced by the CRPD was identified specifically in Lithuania [24], Ireland [27,28], Switzerland [25], Northern Ireland [26], Australia, and Canada [23]. Additionally, case law in England has been directly guided by the CRPD [33]. These changes suggest that within the CRPD’s signatory countries, there is a general movement towards legislating for supported decision-making and the recognition of legal capacity for all. Despite this trend, the mechanisms for implementing such changes in social work practice remain widely varied and their effectiveness under-researched.

There is evidence that despite the shared values of social work and the CRPD and the trend towards legal reform, social work practice itself may be slower to adapt. Shik Kim [35] indicated how the CRPD could be harnessed to strengthen rights-based social work practice and the value of the concept of citizenship. One reason why this does not appear to be happening sufficiently is that local policies and practices are slow to shift. The evidence suggests that sufficient resourcing, together with an active programme of implementation to alter professional cultures, in addition to legal reforms, are required.

Another possibility is that social workers themselves are resistant to changing their practice and approach, despite the legislative context they work within. Based on their research in Israel, Holler and Werner [37,38] conclude that social workers are largely maintaining their modes of practice, despite the legislative changes that have occurred. As indicated, the authors found that practitioners in their studies were largely accepting of guardianship measures and sceptical of supported decision-making, raising questions about their underlying attitudes regarding the rights of persons with disabilities. Further research by the same authors with social work students provides additional evidence of widespread acceptance of social control [41].

The supposition that social work attitudes reflect an acceptance of the status quo is echoed by Maylea [40], who argues that mental health social workers have a clear record of supporting compulsory treatment despite their professional values that, he proposes, challenge it. Maylea indicates that mental health social workers continue to justify involuntary treatment in order to protect the person’s well-being and the wider community; both arguments he rejects wholly. Harding and Taşcıoğlu [36] also demonstrate that social workers default to the best interest principle regularly in England and Wales, despite the Mental Capacity Act’s endorsement of supported decision-making. 

Interestingly, the clearest example in the included literature of changing practice may not be directly linked to the legislative context at all. The innovative case studies in Spain provided by Quejido-Molinero and Miranda-Ruche [32] make it clear that the theoretical roots planted by the CRPD may already be bearing fruit in some practice settings; however, the authors make no note of the Spanish legislation that pre-empted this shift. Instead, they cite the advocacy work of parents of children with intellectual disabilities as having the largest impact on service provision in this area. 

The other example of actual practice was provided by Wilkins [33], who offered a case study in England. The CRPD impacted how a practitioner navigated an ethical dilemma, despite not having directly influenced the legislative framework in place. 

### 4.2. The Benefit of Changing Perceptions

Given that legislative reform is seemingly not having a strong enough impact on social work practice, many are questioning what would result in a greater shift. Holler and Werner [37] have suggested that social workers are slow to reduce their use of substitute decision-making due to a lack of awareness and a lack of availability of more supportive alternatives. To address this gap, they suggest more training on national legislation, and the CRPD may be necessary, albeit Harding and Taşcıoğlu [36] provide one example where practitioners do not offer full support for decision-making despite having received training. However, other evidence in the UK suggests that more in-depth, bespoke training is required. It highlights issues with social workers’ (and other professionals’) knowledge of mental health and capacity legislation despite the relevant statutes being in existence for some time, leading in some cases to unlawful deprivations of liberty and practice that are not compliant with legal principles [44,45]. 

Moreover, several of the included articles point toward not only awareness of the legislative context but also the underlying perception of social workers as a key factor. Much of the literature recognised that the CRPD espouses the social model of disability, which, as indicated, problematises society, rather than the medical model, which locates the ‘impairment’ in the individual [28,43]. Werner and Holler’s [41] research into the underlying attitudes of social work students specifically investigates their perception of disability as either from a social or an individual perspective. Their findings demonstrated a positive link between an individual perspective on disability and an acceptance of guardianship generally. While this research established a relationship between an attitude regarding disabilities and a practice approach, Werner and Holler did not offer a method for changing the perception held by students. Here, we can draw from the study provided by Katsui et al. [42], who tracked the effectiveness of a training scheme that specifically taught the social model of disability. The results indicated that the training was successful in ‘dramatically’ changing the lecturers’ perceptions towards disability, despite their previous deeply held and culturally endorsed views (p. 183). 

While there is evidence that focused training on the social model of disability is effective in changing attitudes, it is questionable if training a relatively limited group (such as social workers) could have an effect on wider practice, for example, on other social care providers or professions. That said, Flynn [28] argues that the public perception of disability in Ireland is changing towards the social model of disability as a result of key stakeholders shifting their own views first. This indicates that changing the attitudes of those with influence may, in time, help to change others. 

### 4.3. The Barrier of Limited Resources and the Need for Systemic Change

In addition to the underlying attitudes of social workers, some have pointed out that the local services themselves, including resourcing and practice cultures, require a significant shift before social work practice may be able to follow suit. Holler and Werner [37] found that one justification given by social workers for the continued use of guardianship orders was that many service providers (particularly in residential settings) require it to be in place, despite the individual’s preferences. Other literature not included in the study’s sample would suggest that a similar dynamic is at play for mental health social workers when making decisions about the use of legal coercion, for example, regarding a lack of alternatives to hospital-based treatment [46]. 

Within the literature, there was reference to positive progress in some places, including in Switzerland [25]. However, it was noted that change was limited by the State’s obligation to maintain existing services, making it difficult to fund the implementation of new ones. In Lithuania, Levickaitė and Mataitytė-Diržienė [24] make a similar point, criticising the local municipality for not redirecting resources despite national legal reform, resulting in largely unchanged service provision. 

The limitations placed on social work practice by a dearth of empowering services were echoed by Ruškus et al. [39], who focus on the ‘role conflict’ created when professional values clash with the requirements of agency policy. This endorses the argument provided by Curto and Marchisio [34], who emphasise the importance of changing wider systems and approaches and not simply relying on workers to adjust individual practice methods. 

The lack of suitable services also points to a wider problem facing the implementation of the CRPD. Despite its legal implications for countries that ratify it into law, the CRPD is powerless to dictate funding and other types of resources. And, while the intention of lawmakers may be to uphold the CRPD by formulating new types of legislation, noticeable change in practice is unlikely to occur without the provision of suitable resources, financial or otherwise. 

Interestingly, the findings of the recent Scottish Mental Health Law Review [16] offer a detailed argument and set of proposals for how the CRPD and broader human rights law can and should be marshalled in addressing this issue. Specifically, it recommends putting economic, social, and cultural rights at the heart of the reform of mental health and capacity law. In alignment with the CRPD, it argues that human rights for people with a mental and intellectual disability regarding legal capacity and coercion cannot be tackled in isolation and can only be realised if commitment is given to meeting a wider range of needs that improve people’s quality of life and address the social determinants of mental impairment. This, it says, necessitates whole systems change, including the use of a human rights framework to inform governmental budgeting decisions, the setting of minimum core obligations to people with mental or intellectual disability (e.g., access to suitable housing, etc.,) and a statutory responsibility on public bodies to secure those aspects of the minimum core obligations reflected in their statutory powers and duties [16] (pp. 171–176). These proposals represent a radical and welcome lever for change, and it will be interesting to see the degree to which they are incorporated in the development of any new Scottish statute(s). 

### 4.4. The Evidence Gap

Lastly, as indicated, this scoping literature review has highlighted a lack of research evidence for the impact of the CRPD on social work practice. This reflects a wider context for social work as an under-researched subject area and the need to build research capacity to find out more about what social workers actually do on the ground and the influences that shape their practice [47]. How the mental health social work role is defined and understood in different jurisdictions is particularly under-researched [48]. With this in mind, the authors are cognisant that mental health social workers, and social workers more broadly, might be developing practice approaches that would fit with the CRPD’s principles but are hidden from the literature. More broadly, we are also aware of initiatives that hold particular interest for mental health social work, including Open Dialogue [49] and Family Group Decision-Making [50], both of which seek to increase autonomy and employ community-based, non-coercive approaches towards improving people’s quality of life and access to human rights. Consequently, we would recommend research that explores these and other innovative social work developments, how they integrate CRPD principles, and what impact they have in effecting meaningful change in people’s lives and upholding their human rights. Relatedly, it would be of value to develop and evaluate the long-term impact of training programmes aimed at improving awareness and professional practice regarding the CRPD, for example, around supported decision-making. Other developments are likely to exist beyond social work practice that align with the CRPD that may not have been researched, or where studies have been undertaken but not yet reported, and it will be of interest to monitor relevant research activity on the back of this review.

## 5. Limitations

Due mainly to resource and time constraints, there were a number of limitations to this scoping review. An inherent limitation of any literature review is the potential to omit relevant sources [22]. To reduce this risk, a robust database search strategy was developed in conjunction with a supervisory panel. Within this, the inability to complete a full hand search of the literature in addition to database searching is a noteworthy drawback. Also, whereas a full systematic review includes an appraisal of the quality of all studies included, a scoping review focuses predominantly on mapping the literature and evidence; the lack of quality appraisal is thus another limitation. Furthermore, the criteria for this review restricted results to those published in English, and it did not include grey literature. Overall, while the review provides valuable insight into the interrelationship between the CRPD and social work practice, its findings need to be viewed in relation to its limited scope together with the context- and location-specific nature of the studies included.

## 6. Conclusions

This review set out to explore the question: ‘What impact, if any, has the CRPD had on social work? A preliminary discussion with practitioners and academics was followed by a scoping literature review, which set out to map the existing literature and was the first time that such a review had been undertaken. The findings have important implications for research, teaching, and practice within the social work field, particularly mental health and adult social work. 

There is a strong link between the tenets of the CRPD and the values of social work, but the review has revealed that there is relatively little empirical research investigating this relationship. The existing literature indicates that the CRPD has influenced legislation relating to social work practice in a number of countries. It also points to some promising developments in terms of the impact on social work practice. This includes innovative service design and practices to enable social workers and care professionals to better manage the challenges and fears associated with supported decision-making. There is also some indication of how the CRPD has positively influenced the research agenda and provided a basis for the development of theory related to human rights and citizenship. 

While these advances are encouraging, the review found more examples of social work practice that appeared not to be aligned with the CRPD, including defaulting to the best interests principles and, in some cases, scepticism towards supported decision-making. These findings question the level of understanding some social workers have about the CRPD and its main tenets. A key recommendation arising from this is the need for focused and high-quality training. This review identified a link between social workers’ underlying perceptions of disability with their approach to practice, in particular, linking the social model of disability with more supportive and less restrictive methods. This suggests that training for social work students and practitioners may be most effective if directed at changing attitudes about disabilities and mental health. Also, the wider literature has indicated problems with social workers’ knowledge of mental health and capacity legislation, which raises significant questions about the legality by which measures restricting autonomy are sometimes being used. This would suggest that dedicated training on the CRPD and relevant national legislation is required, which would include clear guidance on how to implement supported decision-making and how to navigate the challenges of upholding human rights in situations where substitute decision-making and involuntary treatment for mental health appear unavoidable. 

This review also highlights that while the CRPD provides a clear mandate to signatory member states for increasing the understanding of concepts, like legal capacity and upholding the rights, will, and preferences of people with mental and intellectual disability, these aims will not be realised without systemic change to address professional cultures, resourcing, and funding of mental health and other services. It found that practitioners experience ‘role conflict’ between a professional commitment to human rights and local policies and resource constraints that prevent them from practising in ways that are consistent with their values. For this to change, funding and local policy need to be aligned with the CRPD framework. Encouragingly, there is increasing recognition of the need for systemic change towards realising the CRPD’s commitments. This has led to calls to embed economic, cultural, and social rights at the heart of national legislation relating to disability and mental health. This debate offers a welcome context for the development of initiatives in mental health social work and social work more broadly that might help to capitalise on the CRPD’s potential. 

## Figures and Tables

**Table 1:** Search strategy.

*‘* *Convention on the Rights of Persons with Disabilities’*	*(AND)*	*‘Social Work’* *‘Social Services’* *‘Social Care’* *‘Independent Advocacy’*
*In the title, abstract, and keywords (or closest fit)*		

## Data Availability

No new data were created or analysed in this study. Data sharing is not applicable to this article.

## Supplementary Materials

The following supporting information can be downloaded at: https://www.mdpi.com/article/10.3390/ijerph20206927/s1, Table S1: Literature Summary Table.
