# Supplementary material for: The United Nations Convention on the Rights of Persons with Disabilities and Social Work: Evidence for Impact?
Source: Int J Environ Res Public Health. 2023 Oct 16;20(20):6927. doi: 10.3390/ijerph20206927 (PMC10606596; doi:10.3390/ijerph20206927)
Supplement: Supplementary file 1 [file ijerph-20-06927-s001.zip › ijerph-2634277-supplementary.pdf]

Supplementary Table S1 - Literature Summary Table

**Author(s), year Published & country**<br><br>**[numerical reference used in main text]**	**Aims / Purpose & Study Population**	**Study Population & sample size (where relevant)**	**Methods**	**Intervention type / duration / comparator / outcome measures**	**Key Findings that relate to Question**	**Summary / Notes**	**Emerging themes / ideas**
Buschi, Antener & Parpan-Blaser, 2022<br><br>Switzerland<br><br>[25]	Review of Switzerland's policy toward people with ID. Uses CRPD as a standard to drive change	N/A	Review of past and current legislation or service provision policies, as well as research trends within the field	Compares to CRPD Articles	A transition is taking place which normalises care in the community rather than residential setting, however the state is 'bound' to fund the residential locations and therefore struggles to fund alternatives.<br><br>Some progress in changing benefits to allow more autonomy. More research in ID.<br><br>The CRPD is working to motivate and guide policy changes in Switzerland for people with ID, although there is still much room for improvement	No mention of social work, apart from saying they are one group of professionals in this field. Reasonable assumption that SW are involved in the services mentioned, however this is not discussed.	Some positive policy changes but no explicit mention of SW
Campbell, Brophy, Davidson, O'Brien, 2018<br><br>Ireland, Australia, UK, Canada<br><br>[23]	Examines how mental health social workers may provide support for legal capacity in the context of compulsory powers in: UK: Victoria, Australia; Ontario,	N/A	Synthesis of previous literature; explanation and comparison of Mental Health and Capacity laws in 4 distinct countries; use of vignette to discuss the different approaches in	Compare 4 legislative contexts<br><br>Compare one case vignette in 4 locations, discuss legal options and professional roles	Mental Capacity Act (2005) - professionals should enable person to make as many decisions as possible, supported decision-making should be used if possible<br>Mental Capacity Act (Northern Ireland) 2016- crucial to first consider what support might enable him to make relevant decisions<br>Mental Health Act, 2017, Victoria, Australia- encourage voluntary treatment, emphasis on supported	Clear link of legislation which has been influenced by CRPD, thereby influencing and shaping Social Work role.<br>England, NI, and Australia all show clear change to SW role due to new legislation which strives to uphold principles of CRPD. Less clear in Ontario, Canada given no discussion on SDM	Legislation has been impacted. Impact on social workers appears related to nature & timing of legal reform, i.e. implemented post CRPD

	Canada; and Ireland		each country, differing SW role	Focus on rights-based practice is noted	decision-making<br>National Disability Insurance Scheme (2013)- aligns with CRPD, promote human rights and remove barriers to participation. MH Social Work have a large role in this scheme<br>Ontario, Canada- advice from PPAO but no mention of emphasis on Supported decision-making. Social Work role responsible for assessment and post discharge support		
Curto & Marchisio, 2022<br><br>Italy<br><br>[34]	Explores inclusion processes related to the CRPD implementati on process via the '19 Pari' project in Asti (NW Italy) between 2016 and 2020	336 contexts / subjects mapped	Qualitative checklists and surveys used to analyse the work of social workers	Identifying the different phases of the process, highlighting peculiar aspects with respect to the traditional transition paths to adulthood	Shows the necessity to allow interventions that create a support network to achieve full and equal citizenship, rethinking the transition to adult life through new epistemological categories that make it possible to overcome, both in theory and in practice, what is currently defined as a special adulthood	Strong example of possibilities. Clear demonstration of need for service and systemic change.	As opposite
Davidson, Brophy, Campbell, Farrell, Gooding, O'Brien, 2016<br><br>Canada, Australia, England and Wales, and Northern Ireland	Reviews & compares four legal frameworks for supported and substitute decision-making for people with mental health problems	N/A	Compares legislative developments across four chosen jurisdictions	Compares 4 legislative contexts	Helpful overview of legislative change and supported vs substitute decision making.	Good for legal context but no direct exploration of impact on SW practice	As opposite

Doyle and Flynn, 2013<br><br>Republic of Ireland<br><br>[26]	Examines implications of Article 12 of the CRPD which prohibits the removal of a person's legal capacity and instead requires the provision of the supports to ensure exercise of legal capacity basis with	N/A	CRPD articles and concepts		Prior to CRPD, Ireland proposed legislative reform in 2008, The Mental Capacity Bill, which focussed on subs. decision-making and mental capacity, neglecting SDM and legal capacity. These proposals were NOT compatible with CRPD. In 2011, calls were made to introduce reformed legislation in line with CRPD.	Although outdated, this gives a clear picture of how the UNCRPD shaped policy development around capacity.	As opposite.
Flynn, 2021<br><br>Republic of Ireland<br><br>[28]	Critical commentary on 'adults at risk' in safeguarding in Ireland	N/A	Previous literature including policy and theoretical perspectives		Ireland ratified CRPD in 2018. Ireland's legislation has been changing and represents a shift from medical model to social model of disability and more recently to human rights approach	Regarding adult safeguarding, UNCRPD has influenced policy in Ireland, influenced theoretical understanding by key stakeholders and therefore mainstream.<br>No clear link to SW	Legislation has been impacted – also impact on public attitudes following lead of key stakeholders
Harding & Tascıoglu, 2018<br><br>England and Wales<br><br>[36]	Outline key findings from the Everyday Decisions research about how supported decision-making happens in practice	A total of 46 participant s (15 disabled people, 6 supporters, and 25 social care profession als) took part. All	The Everyday Decisions project' involved qualitative interviews with intellectually disabled people and social care professionals with experience of supporting disabled people		Whilst the MCA is a helpful legal framework for supporting everyday preferences, it has not yet been able to catalyse a shift to supported decision-making in relation to wider life choices or complex decisions'<br>-frontline care professionals, many of whom have had significant training in the domestic legal context of the MCA, still default towards best interests, rather than taking the presumption of capacity seriously	Some good SDM exists in daily practice, however further potential and gaps are identified.<br>This paper does NOT compare practice to pre-CRPD and does NOT directly link the CRPD to current practice, however it does compare current policy and practice to the standard set by CRPD	SDM in Practice, although still gaps with potential to fill

		from England and Wales	- discussed their decision-making processes,<br>-interviews transcribed and analysed		-supported decision-making under the CRPD requires something more substantive than the version set out in the MCA<br>- MCA facilitates a lean toward substitute decision making for complex decisions		
Holler & Werner, 2022a<br><br>Israel<br><br>[37]	To examine the meanings social workers attach to guardianship and its alternative, SDM	27 Jewish-Israeli social workers	Semi-structured interviews, conducted by students from university, interviews transcribed and analysed by research team<br><br>Participants asked to elaborate on meaning of Guardianship, SDM and their process of formulating recommendations.	Answers compared to principles of CRPD and literature on SDM and disability rights	Participants felt Guardianship was often necessary; views on SDM varied but most felt it was impracticable and had never put it into their practice; most felt that training was insufficient. Most accepted bureaucratic constraints<br><br>Primary justification for guardianship was the need to protect disabled people from potential risk. 2nd- improve quality of life for person. 3rd- meet third party's needs (service providers)	Demonstrates an academic shift but slow to change policy and practice in Israel. Particularly, clear lack of agreement from social workers about substitute decision-making, indicating CRPD has not yet changed minds of frontline staff. Clear demonstration that public perception and service provision still embrace guardianship, are not accepting of SDM<br>Lack of training to social workers in this area.	Lack of training and awareness in SW on SDM / CRPD principles
Holler & Werner, 2022b<br><br>Israel<br><br>[38]	This study examines how Israeli social workers make legal capacity-related decisions.	27 Social workers (licensed social workers specialising in disability)	Semi-structured interviews, conducted by students from university, interviews transcribed and analysed by research team<br><br>Participants asked to elaborate on	Answers compared to principles of CRPD and literature on SDM and disability rights	Israeli law amended in 2016 to reform Guardianship law and increase protection of vulnerable adults… law adopted the 'necessity' and 'last resort' principles to Guardianship, less restrictive options (SDM) should be taken if possible<br>Social workers continue to link intellectual disability with Guardianship, contrary to principles of CRPD	Laws have been amended in line with CRPD<br>SW practice has not changed<br>Lack of SW awareness of CRPD and practical alternatives to substitute decision making. SW approaches remain same	Amended legislation, SW practice unchanged, lack of awareness within practitioners

			meaning of Guardianship, SDM and their process of formulating recommendations.		Social workers continue to recommend Guardianship due to factors not aligned with CRPD<br>Authors recommend comprehensive training on CRPD and human rights approaches to social workers		
Katsui, Kazakunova& Mojtahedi, 2019<br><br>Kyrgyzstan<br><br>[42]	The main aim of this paper is to tease out the historical and deeply rooted ethical standards, spirituality, and social values that have long supported the social service system in Kyrgyzstan, which, today, faces pressure to align with the Convention	30 university lecturers (trainees) and 6 persons with disabilities (trainers)	The data are based on an intervention programme in Kyrgyzstan between 2017 and 2018 and its follow-up survey.<br><br>30 university lecturers received 2-3 trainings in the framework of the programme and provided feedback after each training.<br><br>Six persons with disabilities were trainers- feedback throughout training and afterwards	Investigates 'traditional' view of disability in Kyrgyzstan and then analyse the transformation of the perception of disability among the university lecturers who received the training<br><br>-social work education is being pushed to align with HR model, but educators still hold historical cultural values, so an EU-SPS programme was provided by Finnish Gov't to retrain them. Delivered in three training	Public services still continue to perpetuate the segregation and isolation of children and adults with disabilities either in institutions or at home, instead of supporting them to be part of society on an equal basis with others.<br>Persons with disabilities are not provided the same opportunities as their peers without a disability, including decision-making. This is largely due to values which perpetuate lower status of persons with disabilities.<br>All of the interviewed social workers and university lecturers confirmed that training on disability in general, and disability rights in particular, is quite rare and very limited. Therefore, they tend to unknowingly reinforce the stigma.<br>After the first trainings, the lecturers' perceptions towards disability changed quite dramatically<br>The outcomes of the EU-SPS verified that such interventions do not automatically translate into a large social change in favour of persons with disabilities	CRPD has directly impacted Social work education in Kyrgyzstan.<br>Training programme has helped to change perceptions of University SW lecturers and therefore future social work students. Established closer links with disability rights advocates.<br>Public policy is not yet changed and therefore change to direct SW practice is more limited, but stigma will be less since training programmes have changed	Changing perceptions in SW educators and potentially next generation of SWs.

				sessions in 2017-2018			
Levickaitė & Mataitytė-Diržienė, 2018<br><br>Lithuania<br><br>[24]	To analyse the Lithuanian social, health care legislation and preparedness of these systems in Vilnius municipality to comply with obligations of Article 19 of CRPD	Lithuanian legislation, policies and practices regarding. Specifically in Vilnius municipali ty	Quantitative and qualitative data was collected from: Websites of Vilnius Municipality, Ministries of Social Affairs and Labour, Health, national NGOs; International and national reports, research and studies; Interviewed representatives of Vilnius Municipality Social Care Department; Questionnaire disseminated among 7 NGOs representing people with disabilities and/or providing services for them in Vilnius city.	Analysed for compliance with Article 19 of CRPD (right to community living and choice, Access to individualised support services, Access to general services,)<br><br>Analysed on structural, process, and output levels	Largely found that this area (Vilnius municipality within wider Lithuanian context) was failing to comply with CRPD Article 19 in every aspect and ill prepared to implement changes Recommendations to move funding away from residential care, change legislation to abolish guardianship, endorse community living and services, provide personal budgets, etc.	Example of how CRPD has not yet changed a country's /city's approach to social care, specifically for people with psychosocial disabilities.<br><br>Focus not on social work specifically but on wider social care law, systems, services. Therefore still relevant.<br><br>Clear example of lack of impact, although they do little to point out positive steps that exist due to CRPD.	Lack of impact, although limited to one location
Maylea, 2017<br><br>Australia<br><br>[40]	To challenge social work's ongoing complacency with	Mental health social work, involuntar y	Drawing on developments in law, psychiatry, psychology, and sociology to argue that social work as	Considers and rejects two justifications for involuntary treatment- being in an	Social work largely continues to uphold the practice of involuntary treatment, which is incongruent with rights-based advocacy and CRPD Author provides alternative approaches to this, citing examples	CRPD has not yet changed Social Work's overarching approach to involuntary treatment, although some examples of positive progress are given	CRPD NOT impacted practice in relation to involuntary treatment

	involuntary treatment	treatment policies and practice	a profession should reject the notion of involuntary treatment	individual's 'best interests', or for the greater good or community protection	such as Sweden's mentor model, or advanced directives. It is unclear if these have resulted because of CRPD		
Ooi & Loh, 2016<br><br>England and Wales<br><br>[31]	This article seeks explore the implications of CRPD and the UK's obligation to eliminate discrimination to persons with disabilities and its obligation to protect the best interests of children.	Parents with disabilities	Case law analysis		Authors identify legal and social mechanisms to support rights and end discrimination of parents with disabilities, in line with CRPD. This includes bolstering current legal processes, such as adding an assessment checklist to provide consistency and monitoring of SW assessments, or introducing social support structures, which effectively enable disabled parents to provide adequate care to their children	An interesting perspective looking at how the CRPD has an effect not only on MH social work or Adult social work, but also on child protection social work. This is particularly linked to CRPD Article 23(2)<br>Does not discuss ways that current practice within SW have adapted due to CRPD; entirely theoretical/recommendation	Addresses impact on children's SW
Quejido-Molinero & Miranda-Ruche, 2019<br><br>Spain<br><br>[32]	To analyse to practice methods for dealing with ethical practice dilemmas arising since the 'paradigm shift' of the CRPD	2 case studies in Spain; one adults with severe mental disability and one with intellectual disability; both in a care home	2 case examples (real world) of strategies which helped practitioners deal with ethical dilemmas in line with rights-based approach; outcomes provided and discussed	1 strategy- professional supervision / group supervision<br>1 strategy - Ethical Reflection Spaces in Social Intervention Services (ERESS)	Both situations were complex and raised anxiety within staff around how to best protect/care for these individuals while respecting their rights and autonomy<br>Both situations involved the staff team working together to reach a balanced conclusion. In these examples each decision upheld the person's rights, but staff no longer felt anxious about allowing them to make their own decision	Great example of CRPD influencing practice, including real world case studies<br>Offers a valuable suggestion that supervision or ERESS model are helpful ways of addressing inevitable ethical dilemmas arising due to rights-based focus	CRPD impacting practice in Spain, valuable case examples given

Ruskus, Kiaunytė, Zaturskis, & Juodkaitė, 2020<br><br>Lithuania<br><br>[39]	To identify main issues that define role conflict for SWs when determining legal incapacity for persons with intellectual or psychosocial disability and to find the way out of the role conflict, suggesting improvements for SW practice	Six social workers of the social services in Klaipėda city	Open-ended interviews with each worker to explore their experience of working with legal capacity and their own views on this.<br>Thematic analysis applied		Three themes identified:<br>Being submerged in pejorative cultural perceptions;<br>Lacking the ability to advocate for a person with intellectual or psychosocial disability;<br>Being doubtful of his/her own mandate, competence and awareness.	As opposite	Illustrates barriers facing progressive, CRPD-informed change.
Shik Kim, 2010<br><br>South Korea<br><br>[35]	Conceptual piece on how CRPD would enable SW to strengthen its human rights commitments.	Social Work profession/ Practice	Analyse tenets of CRPD and Social Work, identify similarities and formulate argument about the opportunity of closer alignment<br><br>Discuss citizenship theory and apply this to work with persons with disabilities	Compare CRPD and Social Work	Social work should consider strengthening the rights-based practice of the profession by referencing it more specifically to the UN (CRPD) and the concept of citizenship as it applies to people with disability.	Strong argument that similarities between Social Work values and CRPD are inherently compatible rather than conflicting; argues that SW should return to rights-based practice and could lean on CRPD (both in theory and to strengthen policy/legal case) to achieve this.	Does not provide any examples of how CRPD affects SW already; mainly focuses on potential
Stevenson, 2010	To explore the CRPD, the Social Model	People with disabilities	Explores personal experience of 'Voices of Change'	Challenges common justifications for	It is possible and beneficial to involve persons with disabilities. Examples	While there was some push to include persons with disabilities in research	Impacting research / inclusion of

Australia<br><br>[43]	of disability, the Emancipatory Disability Research (EDR) paradigm, and the work of other critical researchers in relation to how these might guide the position and practice of the non-disabled researcher		project which involved co-production with people with Down Syndrome	excluding people with disabilities and provides underpinning for why this is their right<br>Poses 'activist researcher' based on 'activist social worker' model, with same principles	given of how to overcome barriers and what benefits were gained.	before, the CRPD has introduced this as one of their human rights and even more emphasis is now on this inclusion.<br><br>No direct data of before/after CRPD but generalities about how thinking has changed for researchers.	persons with disabilities
Sugiura Mahome, Saxena, & Patel, 2020<br><br>Global focus<br><br>[30]	Reviews the impact of current mental health frameworks, rights affected by the practice of involuntary treatment, and describes some noncoercive alternatives	All countries, focus on 177 signatories of CRPD	Review of mental health legislation existing and newly formed, discussion of use of involuntary treatment policies / alternatives	Compares common mental health laws which include the use of involuntary treatment to new strategies which are 'noncoercive'	Examples given of relevant legislation introduced since CRPD- India, BC Canada, China, Costa Rica and Peru<br><br>Ideas for noncoercive alternatives- co-production and community based care.<br><br>NO mention of social work within these examples, apart from noting they may be involved as professionals in 'circle of care' approach.	Review of how CRPD may be impacting legislation and practice of involuntary treatment, although no direct link drawn to social work	As opposite
Werner & Holler, 2020<br><br>Israel<br><br>[41]	Examined the attitudes of Israeli social work students toward guardianship	Participants were 414 undergraduate and graduate level social	Participants completed a structured questionnaire that measured: attitudes toward	Outcome measured = support (agreement / disagreement) of certain ideas.	Social work students tended to support limiting the scope of guardianship, they did not clearly oppose it.<br><br>Attitudes toward guardianship were	Baseline study' of current attitudes of SW students, post CRPD and Israeli law change (endorsing SDM)<br><br>Students still demonstrating	Lack of awareness in SW students, general acceptance of

	and supported decision-making and the relationship between these attitudes and the perceived importance of social work goals, as mediated by perceptions of disability.	work students from Israel	guardianship and supported decision-making, importance of social work goals, and perception of disability. Questionnaire devised by authors. Answers tallied and analysed	Also measured attitudes toward disability as a mediator<br><br>No longitudinal comparison or intervention	positively associated with the individual perceptions of disability (r ¼ 0.41, p < 0.001), and negatively with the social perceptions of disability<br><br>Social model perspective of disability mediated the association between the social work goal of social justice and attitudes toward guardianship	lack of awareness of SDM and general acceptance of Guardianship. Authors claim this will be even stronger in practitioners, however, have not researched practitioners in the same way for comparison	guardianship remains
Wilkins, 2012<br><br>England<br><br>[33]	Discusses ethical dilemmas related to social work practice with young adults with autism, particularly focusing on competing rights of the CRPD	Young adults with autism	Discussion of a case study taken from real life practice, including ethical dilemmas of a social worker's involvement with a young adult who has autism	SW with 21 y/o with autism; competing desires of her parents (caregivers) and the person's desires, as well as the duty of the SW and person's protected rights according to CRPD	The priority is to enable AB to make her own choices<br>From the social worker's point of view, the Convention makes clear that governments have a duty to inform disabled people of their rights and provide them with information about their options and choices.<br>The social worker was also bound to inform AB of all her rights and not just those conducive to persuading AB to agree with the care plan proposed by her parents.<br>Involvement became more successful overall when a trusting relationship was established with individual	CRPD did not change social work values (honesty, transparency, equality) but did give clear duty for person's rights and directive to social worker for how to navigate difficult ethical dilemma<br>Good example of social work practice being impacted by CRPD	CRPD impacting practice (in England) by changing SW duty

Abbreviations used

CRPD: The United Nations Convention on the Rights of Persons with Disabilities
SW: social work
SDM: Supported Decision Making
MH: mental health
ID: intellectual disability
